# Supplementary material for: Towards clinical implementation of artificial intelligence in cancer care: concept mapping analysis of provincial workshop findings
Source: Implement Sci Commun. 2026 Aug 14;7:229. doi: 10.1186/s43058-026-01075-x (PMC13753414; doi:10.1186/s43058-026-01075-x)
Supplement: Supplementary file 3 [file 43058_2026_1075_MOESM3_ESM.docx]

**Supplemental Information. Standards for Reporting Implementation Studies (StaRI) — Populated Checklist**[55,56]

This completed checklist accompanies the manuscript "Towards clinical implementation of artificial intelligence in cancer care: Concept mapping analysis of provincial workshop findings" and is uploaded as Additional file 3. Items in the "Intervention" column are marked N/A because this concept mapping study examines stakeholder perspectives on AI implementation in cancer care broadly, rather than evaluating a specific, evidence-based clinical intervention (EBI).

| **Checklist item** | **Implementation strategy — what to report** | **Reported on page/section** | **Intervention — what to report** | **Reported on page/section** |
| --- | --- | --- | --- | --- |
| 1. Title | Identify as an implementation study; method in title/keywords | Title; Keywords | Identify the EBI | N/A (no single clinical EBI) |
| 2. Abstract | Identify as an implementation study; describe strategy and implementation outcomes | Abstract | Describe the EBI and health outcomes | N/A (no single clinical EBI) |
| 3. Introduction | Problem/gap the strategy addresses | Background | Problem the intervention addresses | N/A (no single clinical EBI) |
| 4. Rationale | Theory/framework and rationale for strategy | Background; Discussion (CFIR) | Rationale/evidence for intervention | N/A (no single clinical EBI) |
| 5. Aims & objectives | Implementation objectives | Background end; Methods (objective) | Intervention objectives | N/A (no single clinical EBI) |
| 6. Design | Design/features; methods standards | Methods: Concept mapping procedure; Analysis | Design/features | N/A (no single clinical EBI) |
| 7. Context | Context (organizational/system) | Methods: Study context (BC Cancer Summit) | Context | N/A (no single clinical EBI) |
| 8. Targeted sites/populations | Sites/personnel/resources; eligibility | Methods: Participants | Population/eligibility | N/A (no single clinical EBI) |
| 9. Description | Description of implementation strategy | Methods: Concept mapping procedure | Description of intervention | N/A (no single clinical EBI) |
| 10. Sub-groups | Sub-groups/nested tasks | Methods: Participants (workshop vs panel) | Sub-groups | N/A (no single clinical EBI) |
| 11. Outcomes | Pre-specified implementation outcomes and assessment | Methods: Analysis | Intervention outcomes and assessment | N/A (no single clinical EBI) |
| 12. Process evaluation | Process objectives/outcomes linked to the mechanism | Methods: Analysis; Results (pattern match) | Process evaluation | N/A (no single clinical EBI) |
| 13. Economic evaluation | Resource use/costs/outcomes methods | Not undertaken (out of scope) | Resource/costs for intervention | N/A (no single clinical EBI) |
| 14. Sample size | Rationale/considerations | Methods: Participants | Sample size | N/A (no single clinical EBI) |
| 15. Analysis | Analysis methods and rationale | Methods: Analysis | Analysis | N/A (no single clinical EBI) |
| 16. Sub-group analyses | A-priori subgroup analyses | Methods/Results: subgroup (clinical vs non-clinical) | Sub-group analyses | N/A (no single clinical EBI) |
| 17. Characteristics | Proportion recruited; characteristics | Results: Workshop outcomes; Statements | Intervention recipients | N/A (no single clinical EBI) |
| 18. Outcomes | Primary/other implementation outcomes | Results: Figures 1–5 | Intervention outcomes | N/A (no single clinical EBI) |
| 19. Process outcomes | Process data mapped to the mechanism | Results (pattern match; subclusters) | Process outcomes | N/A (no single clinical EBI) |
| 20. Economic evaluation | Resource use/cost outcomes | Not undertaken (out of scope) | Intervention costs | N/A (no single clinical EBI) |
| 21. Sub-group analyses | Representativeness and subgroup outcomes | Results: Subgroup analysis | Sub-group outcomes | N/A (no single clinical EBI) |
| 22. Fidelity/adaptation | Fidelity to strategy; adaptations | Methods/Results (standard stages; no deviations) | Fidelity to intervention delivery | N/A (no single clinical EBI) |
| 23. Contextual changes | Context changes affecting outcomes | Discussion/Limitations | Contextual changes | N/A (no single clinical EBI) |
| 24. Harms | Harms/unintended effects | None observed; Results/Discussion | Intervention harms | N/A (no single clinical EBI) |
| 25. Structured discussion | Summary; strengths/limitations; comparisons; conclusions | Discussion; Limitations; Conclusions | Structured discussion | N/A (no single clinical EBI) |
| 26. Implications | Policy/practice/research implications (scalability) | Discussion: Future directions | Implications (sustainability) | N/A (no single clinical EBI) |
| 27. Statements | Ethics; registration; protocol; funding; conflicts | Declarations (UBC REB H24-03306; data/code availability) | Statements | N/A (no single clinical EBI) |
